# Obesidade, Sobrepeso, Adiposidade Corporal e Risco Cardiovascular em Crianças e Adolescentes

**DOI:** 10.36660/abc.20200540

**Published:** 2020-08-19

**Authors:** Weimar Kunz Sebba Barroso, Ana Luiza Lima Souza

**Affiliations:** 1 Universidade Federal de Goiás Goiânia GO Brasil Universidade Federal de Goiás - Liga de Hipertensão Arterial, Goiânia, GO - Brasil; 2 Universidade Federal de Goiás Programa de Pós-Graduação em Ciências da Saúde Goiânia GO Brasil Universidade Federal de Goiás - Programa de Pós-Graduação em Ciências da Saúde, Goiânia, GO – Brasil

**Keywords:** Criança, Adolescente, Hipertensão, Diabetes Mellitus, Síndrome Metabólica, Sobrepeso, Obesidade, Fatores de Risco, Saúde Pública

A obesidade e o sobrepeso são considerados um problema de saúde pública global e que contribuem fortemente para várias doenças crônicas não transmissíveis (DCNT), dentre elas a síndrome metabólica, *diabetes mellitus* (DM), doenças cardiovasculares (DCV) e câncer. São estimados mais de 1.9 bilhões de adultos com sobrepeso, o que representa 39% da população mundial, e 13% de adultos obesos. A Organização Mundial da Saúde estimou, para 2019, mais de 38 milhões de crianças abaixo dos cinco anos com sobrepeso ou obesidade. A obesidade na infância está associada a maior chance de morte prematura, aumento do risco de hipertensão arterial, DM e câncer. Além disso, crianças obesas apresentam marcadores precoces de DCV, aumento do risco de fraturas, dificuldades respiratórias e resistência à insulina.^1^

A obesidade, como fator de risco independente para doenças cardiovasculares,^2^ está relacionada com a elevação dos níveis de apolipoproteínas B (ApoB) e consequente disfunção endotelial. A presença de obesidade e dislipidemias durante a infância reflete o desenvolvimento de morbidades cardiovasculares na idade adulta.^3^

O excesso de adiposidade corporal está relacionado com a presença de dislipidemia, identificada a partir do aumento dos níveis de colesterol total sérico e lipoproteínas de baixa e alta densidade. Ainda, entendendo que a dislipidemia aterogênica e doença aterosclerótica podem ter o seu início na infância e podem estar acompanhadas da obesidade, elas deve ser analisadas como fatores de risco associados à presença da doença coronária (DAC) na fase adulta.^2 , 4 - 6^

Altas concentrações de ApoB e baixas concentrações de ApoA1 têm sido identificadas como marcadores bioquímicos para aterosclerose mesmo em idades mais precoces,^7^ estando associadas com circunferência da cintura, adiposidade e história familiar de DAC.^8^

As apolipoproteínas A1 e B são proteínas essenciais para o metabolismo das partículas de lipoproteínas e seus níveis séricos são reconhecidos como preditores de risco para doença aterosclerótica. A avaliação dos níveis plasmáticos pode auxiliar na identificação do aumento do risco e adoção de estratégias de intervenção precoce. Têm, portanto, a capacidade de adicionar informações clínicas que vão além daquelas obtidas pela avaliação do LDL e HDL.^9 , 10^

Em adultos, as altas taxas de ApoB estão associadas fortemente com a síndrome metabólica e obesidade e são melhores preditoras de risco cardiovascular que as medidas tradicionais de lipídeos sanguíneos. Na população jovem, o perfil lipídico convencional não é um bom preditor de DAC na idade adulta.^7 , 10 - 12^

Na revisão sistemática “Adiposidade corporal e apolipoproteínas em crianças e adolescentes: metanálise de estudos prospectivos”,^13^ a ApoB foi registrada como um marcador cardiometabólico associado com a massa corporal entre adolescentes e crianças, indicando alteração no perfil das apolipoproteínas nessa população.

A relevância desse estudo^13^ pode ser destacada não só pelo achado clínico, definindo relações entre morbidades e biomarcadores, mas também pelo fato de ter sido direcionado para a população de crianças e adolescentes. Os resultados sensibilizam para a necessidade de estratégias de enfrentamento coletivo para problemas de magnitude global como a obesidade e as doenças cardiovasculares.

A inclusão das apolipoproteínas na avaliação padrão do perfil lipídico, como biomarcadores sensíveis para a identificação de risco, pode ser útil como estratégia de rastreamento e detecção precoce, além da construção de indicadores para a vigilância da saúde nessa população.
