# Supplementary material for: Obesidade, Sobrepeso, Adiposidade Corporal e Risco Cardiovascular em Crianças e Adolescentes
Source: Arq Bras Cardiol. 2020 Aug 19;115(2):172–3. [Article in Portuguese] doi: 10.36660/abc.20200540 (PMC8384296; doi:10.36660/abc.20200540)
Supplement: Translation

# Translation: Obesity, Overweight, Body Adiposity and Cardiovascular Risk in Children and Adolescents

**Authors:** Weimar Kunz Sebba Barroso, Ana Luiza Lima Souza

**Affiliations:** 1 Brazil Universidade Federal de Goiás - Liga de Hipertensão Arterial, Goiânia, GO - Brazil; 2 Brazil Universidade Federal de Goiás - Programa de Pós-Graduação em Ciências da Saúde, Goiânia, GO – Brazil

**Keywords:** Child, Adolescent, Hypertension, Diabetes Mellitus, Metabolic Synrome, Overweight, Obesity, Risk Factors, Public Health

Obesity and overweight are considered a global public health problem and contribute strongly to several chronic non-communicable diseases (NCDs), including metabolic syndrome, diabetes mellitus (DM), cardiovascular diseases (CVD) and cancer. More than 1.9 billion overweight adults are estimated, representing 39% of the world population, and 13% of obese adults. The World Health Organization estimated, for 2019, more than 38 million overweight or obese children under the age of five. Childhood obesity is associated with higher chances of premature death, increased risk of high blood pressure, DM and cancer. Besides, obese children have early CVD markers, increased risk of fractures, breathing difficulties and insulin resistance.^1^

Obesity, as an independent risk factor for cardiovascular diseases,^2^ is related to increased levels of apolipoproteins B (ApoB) and consequent endothelial dysfunction. The presence of obesity and dyslipidemia during childhood reflects the development of cardiovascular morbidities in adulthood.^3^

Excess of body adiposity is related to the presence of dyslipidemia, identified from the increase in the levels of total serum cholesterol and low and high-density lipoproteins. Also, assuming that atherogenic dyslipidemia and atherosclerotic disease can start in childhood and be accompanied by obesity, they must be analyzed as risk factors associated with the presence of coronary heart disease (CAD) in adulthood.^2 , 4 - 6^

High concentrations of ApoB and low concentrations of ApoA1 have been identified as biochemical markers for atherosclerosis even at earlier ages,^7^ being associated with waist circumference, adiposity and family history of CAD.^8^

Apolipoproteins A1 and B are essential proteins for the metabolism of lipoprotein particles and their serum levels are recognized as risk predictors for atherosclerotic disease. Evaluation of plasma levels can help to identify increased risk and to adopt early intervention strategies. Therefore, they can add clinical information that goes beyond that obtained by the evaluation of LDL and HDL.^9 , 10^

In adults, high rates of ApoB are strongly associated with metabolic syndrome and obesity and are better predictors of cardiovascular risk than traditional blood lipid measurements. In the young population, the conventional lipid profile is not a good predictor of CAD in adulthood.^7 , 10 - 12^

In the systematic review “Body adiposity and apolipoproteins in children and adolescents: meta-analysis of prospective studies”,^13^ ApoB was recorded as a cardiometabolic marker associated with body mass among adolescents and children, indicating changes in the profile of apolipoproteins in this population.

The relevance of this study^13^ is due not only to the clinical finding, defining relationships between morbidities and biomarkers, but also to the fact that it was aimed at a population of children and adolescents. The findings raise awareness of the need for strategies for collective coping of problems of global magnitude, such as obesity and cardiovascular diseases.

The inclusion of apolipoproteins in the standard evaluation of the lipid profile, such as sensitive biomarkers for risk identification, can be useful as a screening and early detection strategy, in addition to the development of indicators for health monitoring in this population.
